# Construction and Applications of Rabbit Hemorrhagic Disease Virus Replicon

**DOI:** 10.1371/journal.pone.0060316

**Published:** 2013-05-08

**Authors:** Binbin Wang, Mingjia Zhe, Zongyan Chen, Chuanfeng Li, Chunchun Meng, Miaotao Zhang, Guangqing Liu

**Affiliations:** 1 Shanghai Veterinary Research Institute, CAAS, Shanghai, China; 2 College of Veterinary Medicine, Northwest A&F University, Yangling, Shaanxi, China; International Centre for Genetic Engineering and Biotechnology, Italy

## Abstract

The study of rabbit hemorrhagic disease virus (RHDV) has long been hindered by the absence of an in vitro culture system. In this study, using RHDV as a model, a series of DNA-based reporter replicons were constructed in which the firefly luciferase (Fluc) gene was fused in-frame with the open reading frame of the replicon. In this construct, the Fluc gene was inserted where the coding region of viral structural protein was deleted and was under the control of a minimal cytomegalovirus (CMV) immediate-early promoter. Fluc activity analysis showed that these reporter replicons replicate efficiently in mammalian cells. On the basis of the replicon, 5′non-coding regions (5′NCR) and genome-linked protein (VPg) were deleted, and the effect on the expression of replicon was analyzed. The results showed that the expression level of Fluc was reduced in the absence of 5′NCR and VPg, suggesting that the 5′NCR and VPg may play an important role in replication and/or translation of RHDV. To further verify the speculation, we also constructed a replication deficient mutant (pRHDV-luc/Δ3D), and the impact of 5′NCR and VPg deletion on viral translation efficiency was analyzed, our results indicated that both VPg and 5′NCR were involved in RHDV translation.

## Introduction

Rabbit hemorrhagic disease (RHD) is a highly infectious and fatal disease caused by rabbit hemorrhagic disease virus (RHDV), which is the prototype strain for the genus *lagovirus* in the family *Caliciviridae* [1]. The genome of RHDV is composed of 7437 nucleotides (nt) of single stranded, positive sense RNA and encodes two slightly overlapping open reading frames (ORFs): ORF1 and ORF2. ORF1 encodes a polyprotein that produces several non-structural proteins and the capsid protein VP60. ORF2 encodes the minor structural protein, VP10. The genomic RNA is polyadenylated at the 3' end and at their 5′ region it is covalently linked through a Tyr-21 residue to the VPg (virus genome -linked ) protein [2], [3], [4].

The lack of a susceptible cell line or an animal model for RHDV infection has prompted the development of alternative strategies to study RHDV. Vectors based on self-replicating RNAs (replicons) of positive strand RNA viruses are powerful tools for gene expression in mammalian cells. Since their initial development, replicons have been beneficial for studying the life cycle of viruses. Replicons have been described for several caliciviruses, including NV [5] and RHDV [6]. In previous work, we established a full-length infectious cDNA clone of the RHDV strain CHA/JX/97 and DNA-launch-based RHDV reverse genetics system [6], [7]. Depending on the system, we demonstrated that VP2 is not essential for RHDV infectivity_._ In this study, we report a novel RHDV replicon system in which a reporter gene (firefly luciferase, Fluc) replaces most of the coding regions of the structural proteins (VP60 and VP10) and is capable of self-replication in RK13 cells.With the help of the replicon, we also analyzed the effects of the deletion of 5′NTR, 3D and VPg on the expression of viral genome.

## Materials and Methods

### Cells and Viral cDNA

A rabbit kidney cell line (RK13, ATCC NO: CCL-37) was maintained in minimal essential medium (MEM) with 10% fetal bovine serum (FBS) in 5% CO_2_ at 37°C. The parental RHDV strain CHA/JX/97 was isolated from rabbits in Zhejiang (china) during the 1997 out-break. Viral cDNAs were generated and described in our previous work [7].

### Gene Constructs

**Figure 1:**
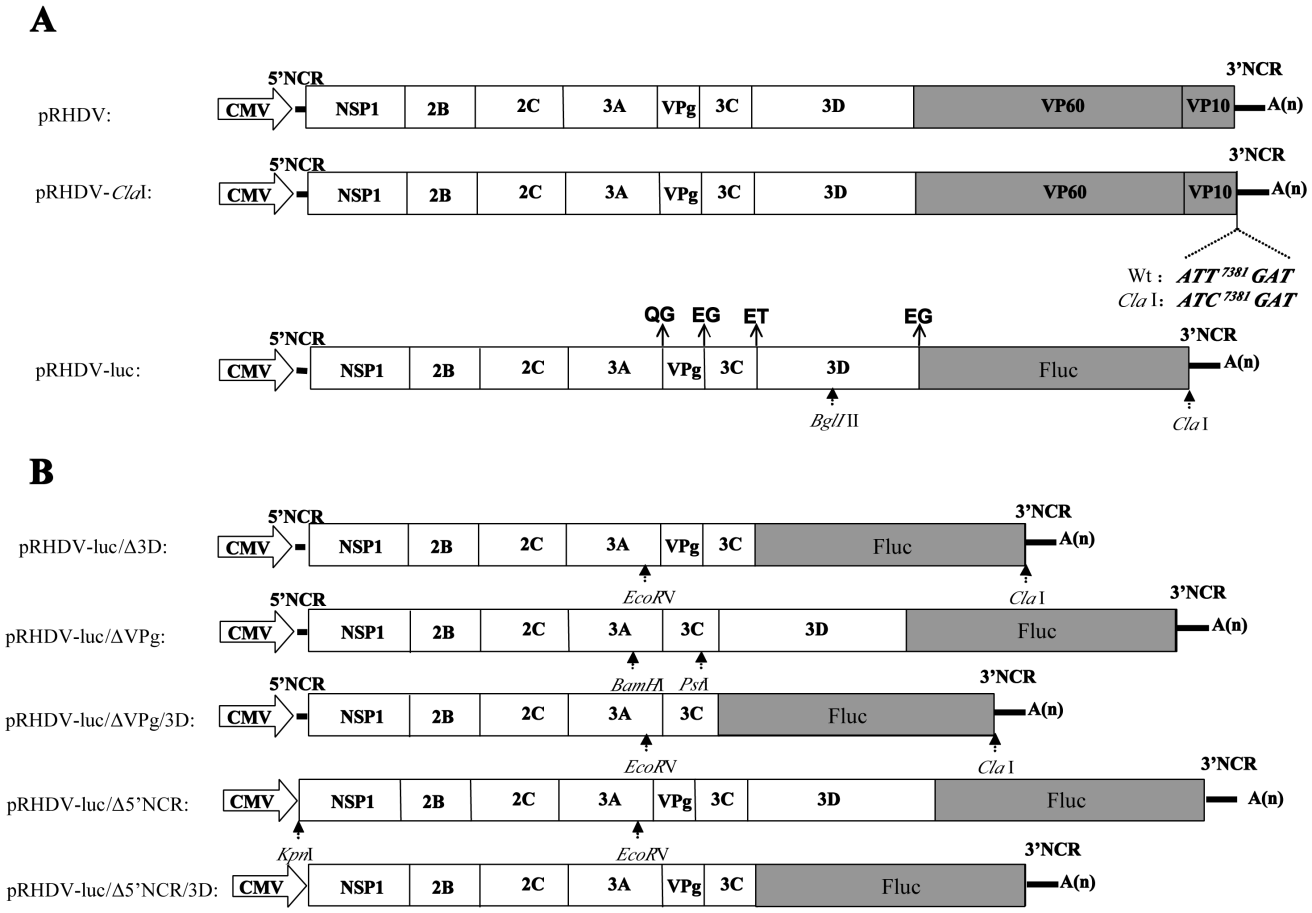
Schematic diagrams of the organization of RHDV luciferase replicon and the derived RHDV deletion mutants. (A) In bold, a schematic representations of RHDV luciferase replicon constructs; the unique *Cla*I site was introduced at 7381 nt. Fluc replaced most of the viral structural protein coding region by fusion PCR. White boxes denote RHDV nonstructural protein coding sequence. Gray boxes indicate a structural protein coding sequence. Lines indicate the 5′ and 3′ NCRs. Arrows point to the positions of the cleavage sites at the N and C termini of the proteins. (B) In-frame deletions of RHDV plasmids were generated by fusion PCR. The deleted sequence and positions are indicated for each mutant. Arrows point to the positions of the restriction site used to construct deletion mutants.

The pRHDV contained the full-length genome of RHDV and was generated in our previous work. To facilitate construction of the RHDV luciferase replicon, a unique *Cla*I restriction site was introduced into pRHDV by standard site-directed mutagenesis. This restriction site was used to insert the PCR product of the firefly luciferase gene, which was amplified from pGEM-LUC (Promega, USA ) by using a set of specific primers (Fluc-F and Fluc-R) (Table 1). The final fusion PCR product, amplified using the primer set F-4843, R-5305, R-6969, was substituted for the *Bgl*II-to-*Cla*I region in pRHDV, and the Fluc gene was inserted in-frame with the start codon of VP60 to replace VP60 gene and part of VP10 (from 5311 nt to 7372 nt) (Figure 1). This plasmid was named pRHDV-luc. To construct the VPg deletion mutant of pRHDV-luc, An in-frame deletion of nucleotides (from 2983 nt to 3327 nt) in the RHDV genome was engineered into the backbone of pRHDV-luc (Figure 1). To do this, a *BamH*I*-Pst*I fragment from pRHDV-luc with a VPg deletion was constructed using standard overlapping PCR. The PCR product was then inserted into pRHDV-luc, which had been digested with *BamH*I/*Pst*I, and the resulting plasmid was designated pRHDV-luc/ΔVPg. Using a similar strategy, A *KpnI* restriction site was ligated to the first nucleotide of the 5'NCR, 5'NCR was deleted from *KpnI*-to-*EcorV* region in pRHDV-luc, the final products was digested with *KpnI/EcorV* after purification and was ligated into the backbone of the pRHDV-luc, pRHDV-luc/Δ5′NCR was constructed. The pRHDV-luc/Δ3D, pRHDV-luc/ΔVPg/Δ3D,and pRHDV-luc/Δ5′NCR/Δ3D were construced using fusion PCR, In-frame deletion of nucleotides (from 3768 nt to 5305 nt) in RHDV genome was engineered into replicons to generate deletion mutants pRHDV-luc/Δ3D, pRHDV-luc/ΔVPg/Δ3D,and pRHDV-luc/Δ5’NCR/Δ3D (figure.1B), the final fusion PCR products were substituted for the EcoRV-to-ClaI region in the pRHDV-luc, pRHDV-luc/ΔVPg and pRHDV-luc/Δ5′NCR replicons. The primers used to generate these deletion mutant are listed in Table 1. All of the replicon cDNA plasmids were verified with restriction mapping and DNA sequencing analysis.

**Table 1:** Oligonucleotides used for construction of RHDV luciferase replicons, VPg deletion mutant, 3D deletion mutant, 5′NCR deletion mutant, and detection of replicon.

Primer	Sequence*(5′→ 3′)	Polarity
**Primers for pRHDV-luc constructs**		
F-4843	ACTCCGATGATGGTGAGCCTT	+
R-5305	TTTTTGGCGTCTTCCATGCGGGTTTTGCCCTCCAT	−
Fluc-F	ATGGAAGACGCCAAAAACATA	+
Fluc-R	AAATCGATTCACACGGCGATCTTTC	−
R-6969	CCAATAAAATCGATTCACACG	−
**Primers for detection of replicon**		
jdfluc-F	TTCGGTTGGCAGAAGCTATG	+
jdfluc-R	GGTAGGCTGCGAAATGCCCA	−
GAPDH-F	AGGGCTGCTTTTAACTCTGGTAAA	+
GAPDH-R	CATATTGGAACATGTAAACCATGTAGTTG	−
jdNSP3-F	GTTATTGGTGCCATCCGAAC	+
jdNSP3-R	GTGTAAGTCACTCACAACCT	−
**Primers for pRHDV-LUC**/**ΔVPg constructs**		
F-BamHI :	TATGGATCCCTGGTTAGA	+
R-2977-2994:	CACGCCCTGGAAGGCTTT	−
F-2980-3009:	AGCCTTCCAGGGCGTGTACGAGGGCCTGCCTGG	+
R-PstI:	CGGCTGCAGACCAAAGTA	−
**Primers for pRHDV-Luc/3D constructs**		
F-EcorV	GGTGATATCACACCTGTG	+
R-3751	TGATGTTTCATAAACTCC	−
F- 5322	GGAGTTTATGAAACATCAATGGAGGGCAAAACCCGT	+
R-ClaI	AAAATCGATTCACACGGCGAT	
**Primers for pRHDV-luc/Δ5’NCR constructs**		
**F-KpnI**	CTAGGGTACCATGCCGCCTATGTC	+

* Underlined nucleotides were required for cloning.

### Transfection

RK13 cells were seeded in 35 mm dishes (200000 cells/well in 2 mL of medium) and grown overnight to approximately 80% confluence. Then, 1 µg of plasmid DNA and a Renilla luciferase plasmid (pGL4.75; Promega) were transfected into cells by effectene transfection reagent (QIAGEN, Germany) following the manufacturer’s protocol, The Renilla luciferase activity was used as an internal control for the normalization of luciferase values obtained from cells transfected with firefly luciferase replicon.

### RT-PCR Analysis

For RT-PCR analysis, 24 h after transfection, total cellular RNA was extracted with Trizol reagent (Invitrogen, USA) according to the manufacturer's instructions and then resuspended in nuclease-free water (Takara, Japan). cDNA was synthesized from 500 ng RNA template using M-MLV reverse transcriptase (Takara, Japan) according to manufacturer's instructions, the cDNA of plus strand RNA was synthesized using the jdFluc-R or jdNSP3-R primer, the cDNA of minus strand RNA was synthesized using the jdFluc-F or jdNSP3-F primer. The control RT-PCR reaction of total RNA from non-transfected RE-13 cells and reaction without the reverse transcriptase (RT) were made, 2 ul of the RT-PCR mixture was added to the PCR reaction.PCR reaction was performed in 35 cycles using Ex Taq polymerase (Takara, Japan). The RT-PCR products were analyzed on 2% agarose gel. The primers used to detection are listed in Table 1.

### qRT-PCR

For RT-PCR analysis, 24 h after transfection, total cellular RNA was extracted with Trizol reagent (Invitrogen, USA) according to the manufacturer's instructions and then resuspended in nuclease-free water (Takara, Japan). RNA samples were treated with DNAse (Takara, Japan) and first strand DNA synthesis was conducted using M-MLV reverse transcriptase (Takara, Japan) according to manufacturer's instructions, with random hexamers using DNAse treated RNAs as templates. 2 ul of the RT-PCR mixture was used in various qRT-PCR reactions using either primers GAPDH-F and GAPDH-R to amplify a fragment of the internal control GAPDH sequence, or primers jdfluc-F and jdfluc-R to amplify the fluc sequence. Triplicates of samples were pipetted into a 96-well qPCR-plate and reactions were performed with SYBR Green PCR mix (Takara, Japan) using an Mastercycler ep realplex real-time PCR machine (Eppendorf, Germany). Three biological replicates were done for each treatment. PCR amplification was performed using a program of 10 min at 95°C followed by 40 cycles of 15 s at 95°C, 30 s at 55°C and 30 s at 72°C. RNase-free water and cDNA synthesized from RNA isolated from noninfected RK13 cell were routinely used as a no-template control for every run. At the end of the assay, the specificity of each amplified product was ascertained by the means of melting curve analysis 15 s at 95°C, 20 s at 60°C and 15 s at 95°C. This eliminated false-positive detections due to primer dimers or nonspecific amplicons.

In preliminary experiments, the PCR products were separated on agarose gels, products were confined to a single band of the expected size for each specific target. The PCR efficiency was >90% in all PCR runs. A comparative threshold cycle (C_T_) was used to determine gene expression relative to the control (pRHDV-luc). For each sample, *fluc* C_T_ value was normalized using the formula △C = C_T(fluc)_ – C_T(GAPDH)_. To determine relative expression levels, the following formula was used: △△_CT_ = △_CT(sample)_ –△_CT(control)_. Relative *fluc e*xpression was calculated using the expression formula 2^−△△ct^.

### Growth Curve in Cell Culture

RK13 cells were grown in 35 mm dishes. The cells were transfected with plasmids DNA and pGL4.75 incubated at 37°C in 5% CO_2_. The cells were harvested at 12, 24, 36, 48, 60 and 72 h post-transfection and stored at −80°C.

### Indirect Immunofluorescence Assay (IFA)

For Indirect immunofluorescence assay (IFA), RK13 cells were fixed in 3.7% paraformaldehyde in PBS (pH 7.5) at room temperature for 30 min and permeabilized by incubation in −20°C methanol for 30 min. The fixed cells were washed with PBS and incubated for 2 h at 37°C with a 1∶1000 dilution of monoclonal antibody specific for Fluc (Promega, USA). After incubation with primary antibody, cells were washed with PBS and incubated for 1 h at 37°C with a 1∶1000 dilution of rabbit anti-goat immunoglobulin G conjugated to fluorescein isothiocyanate. Finally, the samples were observed under a fluorescence microscope equipped with a video documentation system.

### Luciferase Activity Determination

One day after transfection, each well with RK13 cells was washed with PBS, and the cells were lysed in 500 µl of reporter lysis buffer (Promega, USA) per well of a 6-well plate. The cells were then incubated on a shaker at room temperature for 15 min. The content of each well was transferred to a microcentrifuge tube, and the samples were centrifuged at 12,000×g for 2 min at 4°C. Next, 20 µl of the supernatant was assayed for firefly luciferase activity using a dual-luciferase reporter assay system (Promega) according to the manufacturer's instructions. Luciferase activities were measured using a FB12 Luminometer (Berthold,Germany).

## Results

### Construction and Characterization of RHDV Replicon

The overall strategy to construct the pRHDV is outlined in Figure 1. In our previous studies, a full-length cDNA clone of RHDV was constructed, pRHDV was driven by hCMV promoter, and the core sequence of HDV ribozyme was placed immediately downstream of the viral poly (A) tract to ensure that the final transcripts had the correct 3′ end. The pRHDV is capable of self-replication in RK cell [6], [7]. To monitor the replication of the pRHDV and to explore the possibility of expressing foreign genes using the system, we engineered a reporter gene into pRHDV. One common strategy of this replicon is to use a large internal in-frame deletion that corresponds to the removal of most of the structural protein coding regions. For RHDV, retention of a small portion of the structural protein coding sequence has been shown to be essential for autonomous replication. ORF1 encodes a large polyprotein, and it gives rise to mature viral proteins by proteolytic cleavages at its N and C termini and produces several non-structural proteins and the capsid protein, VP60. ORF2 encodes VP10, and the start codon of ORF2 is located at nt 7025 and shares a 17 nt overlap with ORF1 [4]. Thus, for construction of RHDV replicon, the majority of the structural protein VP60 and the VP10 coding sequence was replaced by Fluc. For the replicon to produce mature protein, the viral cleavage sites must be retained for substrate recognition and cleavage. Therefore, we included the coding sequences for the two amino acids at the N termini of the VP60 protein. Using a similar strategy, VPg and 3D deletion mutants retain the two N and C-terminal amino acids as viral cleavage sites to yield functional proteins [8], [9], The positions of the cleavage sites at the N and C termini of the proteins were shown in Figure 1A.

**Figure 2:**
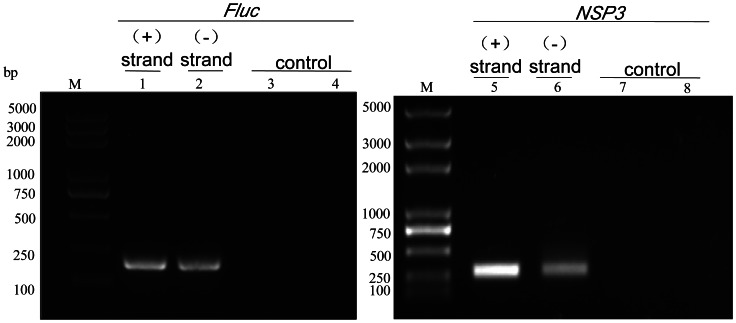
Detection of RNA replication with RT-PCR. Cytoplasmic RNA isolated 24 h post-transfection from RK-13 cells transfected with pRHDV-luc was subjected to standard RT-PCR to detect the accumulation of plus(+) and minus (−) strand. RT-PCR products separated by agarose gel electrophoresis show two PCR products from the pRHDV-luc, Lane M of the gel contains the DNA marker, lanes 1and 2 show RT-PCR reactions of reporter gene *Fluc*, lanes 5 and 6 show RT-PCR reactions of virus gene, lane 3 and 7 are control demonstrating that RT-PCR reactions without reverse transcriptase did not result into amplification of *Fluc* and *NSP3*, lane 4 and 8 show RT-PCR reaction of total RNA from non-tranfected RK-13 cells. Primers are shown in Table 1.

**Figure 3:**
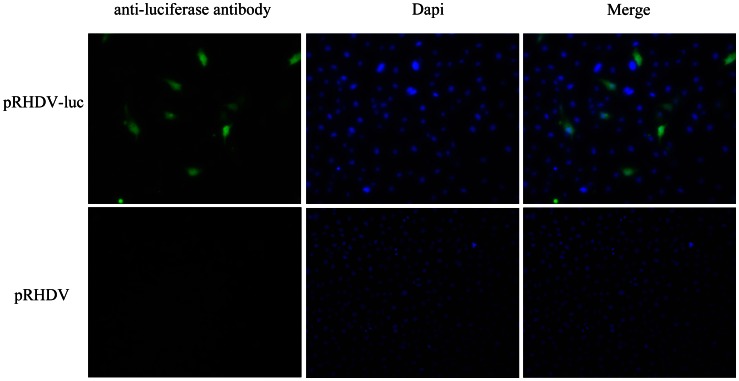
IFA of replicon reporter gene expression in RK13 cells transfected with pRHDV-luc. Detection of Fluc protein in pRHDV-luc transfected cells at 24 h post-transfection by immunofluorescence using a goat polyclonal anti-luciferase antibody. and pRHDV served as a negative control. The Nuclei were stained with DAPI (blue color).

Following the transfection of RK-13 cells with pRHDV-luc, the expression of mRNA was evaluated by performing RT-PCR with RNA samples that were harvested at 24 h post-transfection. The RT-PCR products separated by agarose gel electrophoresis show two PCR products from reporter gene *fluc* and viral gene *NSP3*. Both plus- and minus-strand RNAs of virus were detected. (Figure. 2). IFA of Fluc protein expression in cells transfected with pRHDV-luc was readily detect able in RK-13 cells using a fluc monoclonal antibody (Promage, USA), and the cells transfected with pRHDV failed to cross-react with the Fluc monoclonal antibody (Figure. 3). These results confirmed the ability of RHDV replicon automatically replicate in RK13 cells.

**Figure 4:**
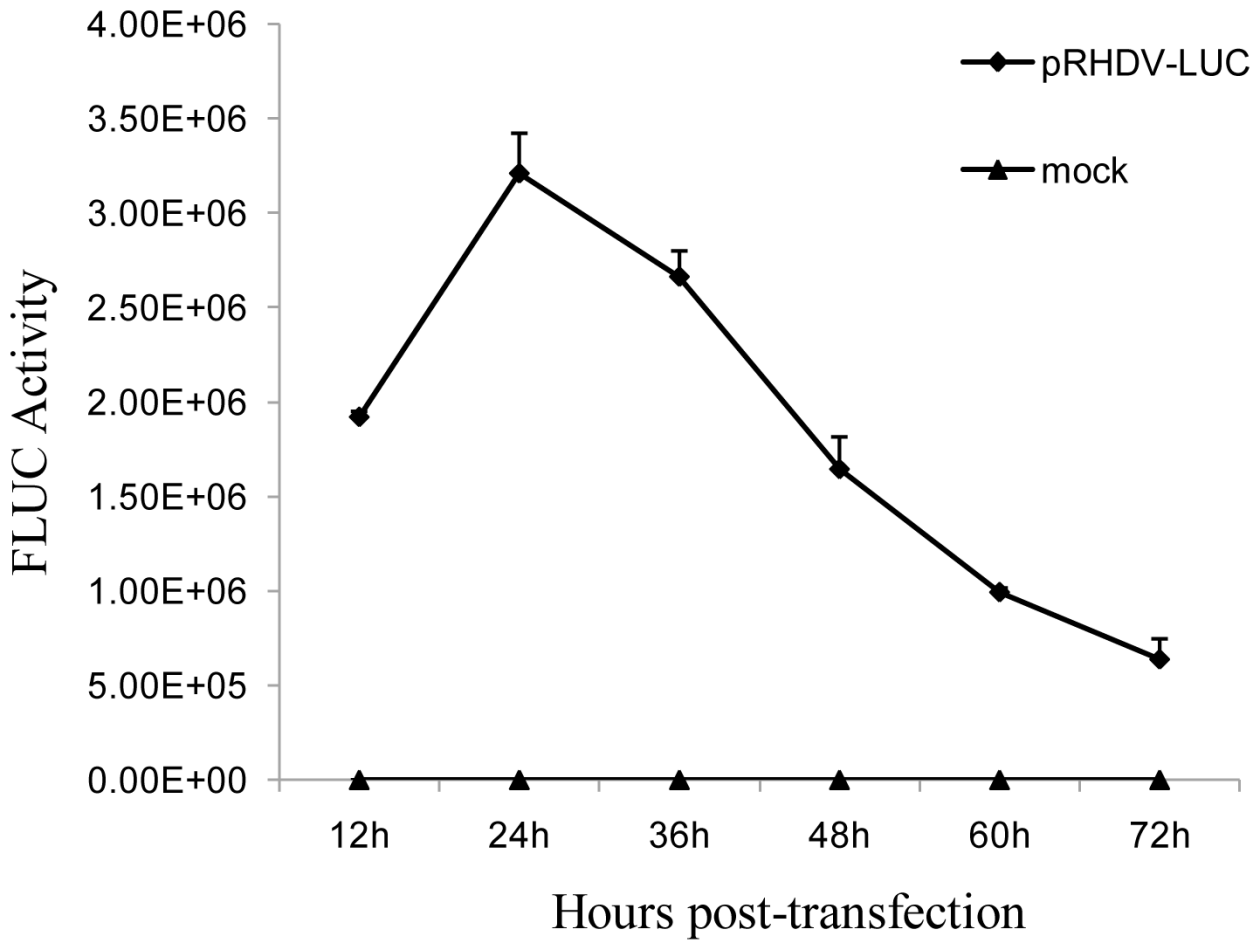
Time kinetics of the replicon in RK 13 cells. During time kinetics with the pRHDV-luc experiments, RK13 cells (200000 per well of a 6-well plate) were transfected with 1 µg of pRHDV-luc and pGL4.75. The cells were then lysed at different times post-transfection, and the Fluc activity was measured in RLU and normalized with respect to a cotransfected plasmid encoding a Renilla luciferase. Similar results were obtained in two independent experiments. pRHDV was used as negative control. In most cases, the variations are very small and therefore the error bars are not visible.

To evaluate the replication dynamics of RHDV replicon, RK-13 cells were transfected with pRHDV-luc and subsequently assayed for fluc activity at 12, 24, 26, 48, 60 and 72 h post-transfection. Fluc activity was normalized with respect to a cotransfected plasmid encoding a Renilla luciferase. Similar results were obtained in three independent experiments. The results showed that the level of Fluc activity in RK13 cells reached a maximum value at 24 h post-transfection and then declined gradually (Figure. 4).

### qRT-PCR Detection of Viral mRNA Levels

**Figure 5:**
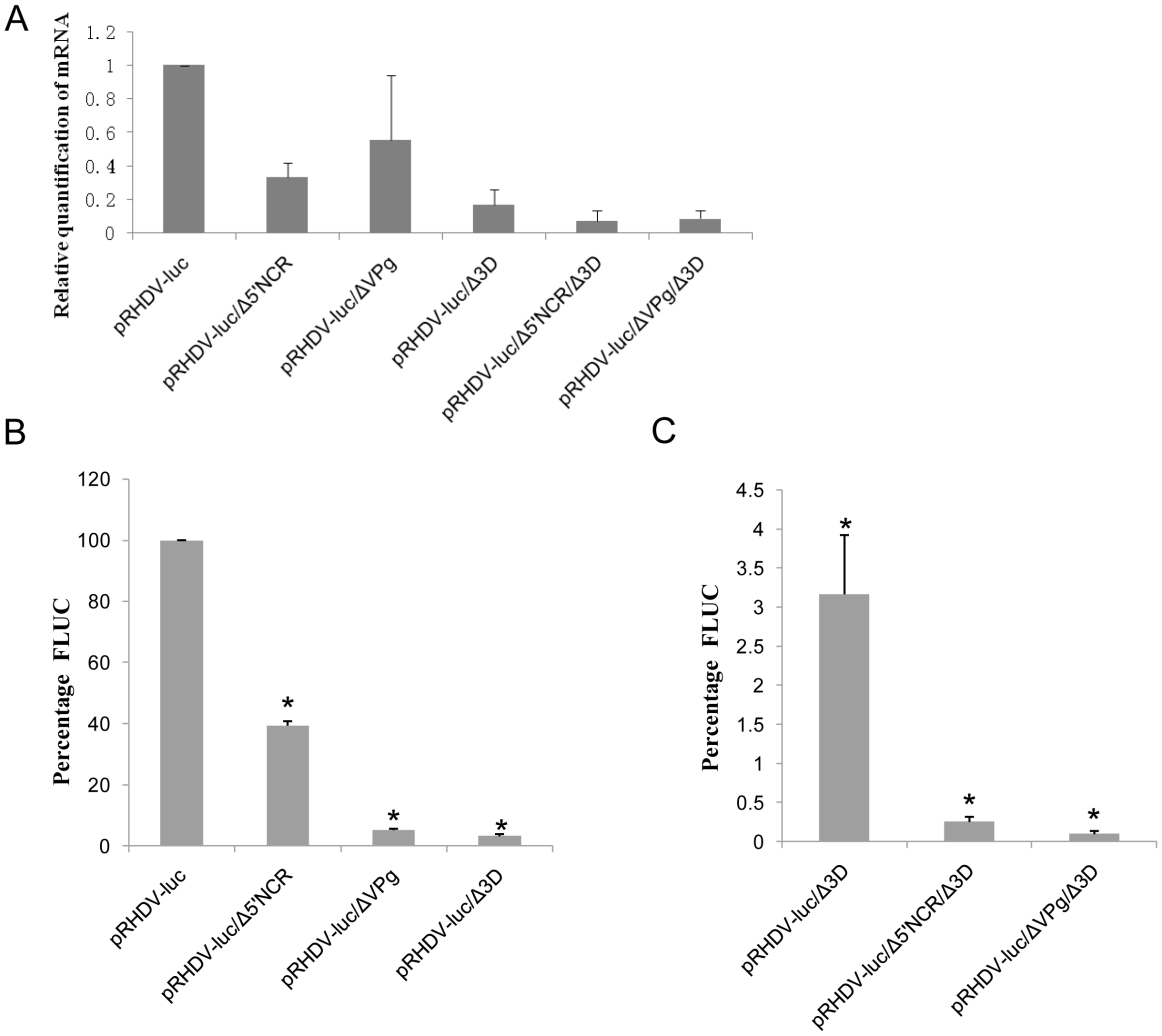
Luciferase activity in cells transfected with the RHDV luciferase replicon and several deletion mutant derivatives and qRT-PCR detection of viral mRNA levels. (A) Relative Luciferase activities in RK13 cell of mutants with deletions in 5'NCR,VPg, 3D, and the parental genotype pRHDV-luc. (B) Relative Luciferase activities levels in RK-13 cells of the replication-deficient plasmid (pRHDV-luc/Δ3D) and its derived deletion mutants (pRHDV-luc/Δ5’NCR/3D, pRHDV-luc/ΔVPg/3D) at 24 h post-transfection.The luciferase activity in RK13 cells was measured by determining the firefly luciferase activity at 24 h post-transfection, and normalization for transfection efficiency was performed by using the Renilla luciferase activity measured at the same time. The differences in luciferase activities produced by the pRHDV-luc and their mutant derivatives were compared by one-way ANOVA and Tukey’s multiple comparison test. Asterisks (*) indicate statistical differences compared to the parental genotype pRHDV-luc. RLU, relative light units.(C)qRT-PCR was used to evaluate the RNA level of viruses derived from pRHDV-luc and their mutant derivatives. The value determined with the parental genotype pRHDV-luc was set as 100% and used as a reference to normalize the replication of the other mutant replicons. Values are means and standard deviations for three independent experiments.

In order to examine how deletion mutants affected *fluc* mRNA levels, we performed qRT-PCR annalysis of RNA isolated from samples transfected with pRHDV-luc and its derived deletion mutants, the results showed that deletion mutations 5′NCR and VPg, which resulted in a decrease of *fluc* mRNA (Figure. 5A). The percentages of *fluc* mRNA levels of replication-deficient pRHDV-luc/Δ3D, pRHDV-luc/ΔVPg/3D, or pRHDV-luc/Δ5′NCR/3D, compared to the pRHDV-luc were approximately 16%,10%,6%. In a word, these findings demonstrate that the 5′NCR, VPg and 3D are important for the production of *fluc* mRNA.

### The Deletions of 5'NCR,VPg and 3D could Reduce the Expression Level of Fluc

To study on the relation between the deletion of 5′NCR,VPg or 3D and RHDV translation and/or replication, a series of in-frame deletion mutants were constructed based in the backbone of pRHDV-luc, equal amounts of parental and mutant replicon plasmids were co-transfected into RK13 cells, Renilla luciferase activity (pGL4.75,Promage, USA) is used to normalize Fluc activity, The effect of the deletions on the expression level of fluc was evaluated by detecting the luciferase activity. The percentages of luciferase activity of mutant replicons pRHDV-luc/Δ5′NCR, pRHDV-luc/ΔVPg and pRHDV-luc/Δ3D, compared to the pRHDV-luc level were approximately 39.4%, 5.2%, and 3.1% (Figure. 5B). The experimental results also showed that fluc activity derived from the VPg mutant was significantly lower than that of parental replicon, which implied that VPg may play a significant role in the life cycle of RHDV. As a control,a null replication mutant of the RHDV was constructed by deleting the *3D* gene (encoding RNA-dependent RNA polymerase, *RdRp*) from pRHDV-luc.

### VPg and 5′NCR are Essential for RHDV Translation

To explore the relation between VPg/5′NCR and viral translation, we performed luciferase activity analysis of RK-13 cells transfected with pRHDV-luc/Δ3D,pRHDV-luc/ΔVPg/3D, or pRHDV-luc/Δ5′NCR/3D, along with pGL4.75(Rluc). As shown in (Figure. 5C), either deletion of VPg or 5′NCR could severely impaired the level of fluc production. To test whether the level of luciferase activity depends on the mRNA concentration, qRT-PCR analysis showed that the levels of *fluc* mRNA of pRHDV-luc/3D, pRHDV-luc/ΔVPg/3D and pRHDV-luc/Δ5′NCR/3D compared to the pRHDV-luc were approximately 16%,10%,6% (Figure. 5A). The results proved the deletion mutants of 5′NCR and VPg could reduce the expression level of Fluc, indicating they were essential for viral transcription and translation.

## Discussion

Reverse genetics of plus-sense RNA viruses using cDNA copies of viral genomes to generate infectious RNA transcripts is a powerful tool to study the molecular details of all aspects of viral life cycles [10]. RHDV is a highly virulent pathogen of rabbits, and the studies into the molecular mechanisms of RHDV replication and translation have been hindered by the lack of an in vitro culture system. To overcome the obstacle, the aim of this study was to establish an experimental system for studying RHDV. We constructed a plasmid containing an *Fluc* gene fused in-frame with the viral ORF in the position where the structural genes were deleted (Figure. 1). The replication of the replicon in transfected RK13 cells was demonstrated by IFA, RT-PCR and qRT-PCR, which monitored the synthesis of viral proteins and replicon RNA. Time course analysis showed that the intensity of the luciferase signal reached a maximum value at 24 h post-transfection. As far as we are aware, this is the first report of such a system developed for RHDV. Using this system, we initiated functional analysis of some important regions of RHDV.

Caliciviruses have some unique properties when compared with other related families of positive-stranded RNA viruses. For example, the 5′NTR of caliciviruses is extremely short and the first ORF of RHDV starts just after nine nucleotides, whose length argues against the present of an internal ribosomal entry side (IRES), besides RHDV and other caliciviruses also *lack* a *5*′*cap* structure at *its terminus*. Therefore, in the Caliciviridae family, recent studies support a novel mechanism of calicivirus VPg-dependent protein synthesis [11], [12], [13]. The role of the calicivirus VPg protein in viral translation was first confirmed for feline calicivirus (FCV). The VPg protein interacts directly with the cap-binding protein eIF4E, and this interaction is essential for viral translation [11], [14]. Thus, the calicivirus VPg protein may function as a proteinaceous cap substitute, where RHDV VPg is covalently linked to the 5′ end of genomic and subgenomic RNA through a Tyr-21 residue that is involved in uridylylation [15]. Additionally, the VPg protein may play a role in replication and function as a protein primer of the viral RNA polymerase [16]. These data indicated that the genome-linked protein VPg in calicivirus infection is multifaceted [17]. In this research, VPg deletion significantly reduced the expression level of the reporter gene, implying that the VPg protein plays a critical role in the life cycle of RHDV. However, the details of its potential role in RHDV replication and translation await further study.

The 5′NCR of RHDV is very short and displays a high degree of conservation [18], we speculate that the 5′NCR may be necessary for virus survival. Additionally, the 5′ ends of the genomic RNA and the subgenomic RNA of RHDV, an extremely high homology was found [19], suggesting that it might be important for virus replication and/or translation [20]. However, in the case of RHDV little is known about the mechanisms of viral replication and translation. In this study,we have utilized the system for analyzing the potential effect of 5'NCR on viral translation. Our results showed that 5′NCR played an important role in viral transcription and translation.

The absence of a permissive cell line and a susceptible animal model for RHDV infection has made it difficult to study the biology of the virus., The results of this study demonstrated that the reporter replicon replicates efficiently in mammalian cells and the expression level of the replicon reporter gene correlates to the level of genome replication and translation compared with the full-length infectious clone. The replicons contain a sensitive reporter gene. The quantitative analysis of luciferase activity is helpful in monitoring the effects of mutations on viral replication and translation. In this study, the luciferase activity was reduced in the absence of 5′NCR, VPg and 3D,and VPg and 5′NCR are involved in viral transcription and translation.

These results provide a basis for further study of the molecular mechanism of RHDV. The availability of the RHDV system that expresses a reporter gene should lead to new insights into RHDV replication and translation.

## References

[1] Lawson M (1995) Rabbit virus threatens ecology after leaping the fence. Nature 378: 531. 8524371 10.1038/378531b0

[2] Cubbitt D, Bradley DW, Carter MJ (1995) Family Caliciviridae. Arch Virol 10: 359–363.

[3] Rasschaert D, Huguet S, Madelaine MF, Vautherot JF (1995) Sequence and genomic organization of a rabbit hemorrhagic disease virus isolated from a wild rabbit. Virus Genes 9: 121–132. 7732658 10.1007/BF01702655

[4] Abrantes J, Loo W, Pendu JL, Esteves PJ (2012) Rabbit haemorrhagic disease (RHD) and rabbit hemorrhagic disease virus (RHDV): a review.veterinary research. 43: 12. 10.1186/1297-9716-43-12 PMC3331820 22325049

[5] Chang KO, Sosnovtsev SV, Belliot G, Adriene DK, Kim YG (2006) Stable expression of a Norwalk virus RNA replicon in a human hepatoma cell line.Virology. 353: 463–473. 10.1016/j.virol.2006.06.006 16843517

[6] Liu GQ, Ni Z, Yun T, Yu B, Chen L, et al (2008) A DNA-launched reverse genetics system for rabbit hemorrhagic disease virus reveals that the VP2 protein is not essential for virus infectivity. Journal of General Virology 89: 3080–3085. 19008396 10.1099/vir.0.2008/003525-0

[7] Liu GQ, Ni Z, Yun T, Zhang YY, Du Q Y, et al (2006) Rescued virus from infectious cDNA clone of rabbit hemorrhagic disease virus is adapted to RK13 cells line. Chinese Science Bulletin 14: 1698–1702.

[8] Wirblich C, Sibilia M, Boniotti MB (1995) ORF1 polyprotein and analysis of cleavage virus: identification of cleavage sites in the 3C-like protease of rabbit hemorrhagic disease. J. Virol 69(11): 7159. 7474137 10.1128/jvi.69.11.7159-7168.1995 PMC189637

[9] Joubert P, Pautigny C, Françoise M (2000) identification of a new cleavage site of the 3C-like protease of rabbit haemorrhagic disease virus. Journal of General Virology 81: 481–488. 10644847 10.1099/0022-1317-81-2-481

[10] Hass M, Gölnitzaff-1 U, Müller S, Becker-Ziaja B, Günther S, (2004) Replicon System for Lassa Virus.J Virol 78.24: 13793–13803. 10.1128/JVI.78.24.13793-13803.2004 PMC533938 15564487

[11] Herbert TP, Brierley I, Brown TD (1997) Identification of a protein linked to the genomic and subgenomic mRNAs of feline calicivirus and its role in translation. J Gen Virol 78: 1033–1040. 9152420 10.1099/0022-1317-78-5-1033

[12] Goodfellow I, Chaudhry Y, Gioldasi I, Gerondopoulos A, Natoni A, et al (2005) Calicivirus translation initiation requires an interaction between VPg and eIF4E.EMBO Rep. 6: 968–972. 10.1038/sj.embor.7400510 PMC1369186 16142217

[13] Daughenbaugh KF, Fraser CS, Hershey JWB, Hardy ME (2003) The genome-linked protein VPg of the Norwalk virus binds eIF3, suggesting its role in translation initiation complex recruitment. EMBO J 22: 2852–2859. 12773399 10.1093/emboj/cdg251 PMC156748

[14] Mitra T, Sosnovtsev SV, Green KY (2004) Mutagenesis of tyrosine 24 in the VPg protein is lethal for feline calicivirus. J Virol 78: 4931–4935. 15078978 10.1128/JVI.78.9.4931-4935.2004 PMC387666

[15] Machin A, Martin Alonso JM, Parra F (2001) Identification of the amino acid residue involved in rabbit hemorrhagic disease virus VPg uridylylation. J Biol Chem 276: 27787–27792. 11369764 10.1074/jbc.M100707200

[16] Han KR, Choi Y, Min BS, Jeong H, Cheon D, et al (2010) Murine norovirus-1 3D^pol^ exhibits RNA-dependent RNA polymerase activity and nucleotidylylates on Tyr of the VPg. J Gen Virol, 91(Pt 7): 1713–1722. 10.1099/vir.0.020461-0 20219896

[17] Goodfellow I (2011) The genome-linked protein VPg of vertebrate viruses –a multifaceted protein. Current Opinion in Virology 1: 355–362. 22440837 10.1016/j.coviro.2011.09.003 PMC3541522

[18] Clarke IN, Lambden PR (1997) The molecular biology of caliciviruses. Gen Virol 78: 291–301. 10.1099/0022-1317-78-2-291 9018049

[19] Meyers G, Wirblich C, Thiel H (1991) Genomic and subgenomic RNAs of rabbit hemorrhagic disease virus are both protein-linked and packaged into particles.Virology. 184(2): 677–686. 10.1016/0042-6822(91)90437-G PMC7131244 1887589

[20] Heinz JT, Matthias K (1999) Caliciviruses: an overview. Veterinary Microbiology 69: 55–62. 10515270 10.1016/s0378-1135(99)00088-7

